# Rechtsstaatlichkeit in der EU als Schlüsselfaktor für eine resiliente Außenpolitik gegenüber Autokraten

**DOI:** 10.1007/s12399-020-00817-6

**Published:** 2020-11-05

**Authors:** Robert Stüwe

**Affiliations:** Zentrum für Europäische Integrationsforschung (ZEI), Universität Bonn, Genscherallee 3, Bonn, Deutschland

**Keywords:** Gemeinsame Außen- und Sicherheitspolitik (GASP), Rechtsstaatlichkeit, Außenhandelspolitik, EU-Erweiterung, Illiberalismus, Energiepolitik, Common Foreign and Security Policy (CFSP), Rule of law, Trade policy, EU enlargement, Illiberalism, Energy policy

## Abstract

Rechtsstaatsfeindliche Regierungsparteien in der EU buhlen um die Gunst autokratischer Großmächte wie China oder Russland und machen die Union so anfälliger für deren Einflussnahme. Das Zusammenwirken undemokratischer Kräfte von innen und außen untergräbt dabei nicht nur den Rechtsstaat, sondern legt auch zentrale außenpolitische Machthebel der EU gegenüber Drittstaaten – die Erweiterungs- und Außenhandelspolitik – lahm. Zudem verfestigen sich staatskapitalistische Strukturen, welche die Funktionsfähigkeit der gemeinsamen Energiepolitik einschränken. Der Beitrag analysiert diese Entwicklungen und erörtert, was die EU der Autokratisierung entgegensetzen kann.

## Abstract (en)

Anti-rule of law governing parties in the EU are courting the favor of autocratic great powers such as China or Russia, making the Union more vulnerable to their influence. This coalition of internal and external autocrats undermines the rule of law and further, the Union’s central foreign policy levers vis-à-vis third countries—enlargement and trade policy—are becoming less effective. In addition, state-capitalist structures are consolidating, which also limits the proper functioning of the EU’s energy policy. This article explores these developments and discusses what the EU can do to counteract autocratization.

## Problemaufriss

Rechtsstaatsfeindliche Regierungsparteien in der EU und autokratische Großmächte wie China und Russland treiben einen Keil in die Europäische Union. Die Bedingungen dafür sind nicht erst seit dem Ausbruch der Covid-19-Pandemie günstig: Seitdem die USA unter Präsident Donald Trump als Schutzmacht der europäischen Demokratieentwicklung an Wirkungskraft einbüßen, haben Peking und Moskau in der EU einen weiteren Handlungsspielraum. EU-Mitgliedstaaten und Beitrittsaspiranten mit *illiberalen* Ambitionen fühlen sich so bestärkt. Das schwächt die außenpolitische Handlungsfähigkeit der Gemeinschaft. Was kann die EU diesem Trend entgegensetzen?

Eine Voraussetzung liegt in der Klärung der heimischen Regimefrage: Denn der Umstand, dass innereuropäische Rechtsstaatsgegner – wie der ungarische Fidesz, die italienische Lega oder die Freiheitliche Partei Österreichs (FPÖ) – die autokratische Herrschaftsideologie aus Peking und Moskau im Grundsatz teilen, nähert auch ihre außenpolitischen Ziele einander an. In der EU entsteht daraus ein Interessengegensatz über die Zweckbestimmung und Strategien der europäischen Außenpolitik zwischen verfassungstreuen Mitgliedstaaten und ihren Gegenpolen. Die Fragen, ob das auswärtige Handeln der Europäischen Union weiter primär dem Zweck der Demokratieförderung dienen soll und ob die EU ihre Marktmacht wie bisher strategisch zur Stärkung multilateraler Institutionen einsetzen darf, werden gegensätzlich beantwortet (Meunier und Vachudova 2018). Dieser doppelte Dissens begünstigt eine Entwicklung, an deren Ende zwei elementare Politikfelder des auswärtigen Handelns unter die Räder kommen. Eine solche Erosion der Funktionsfähigkeit geht über den unmittelbaren Regelungsbereich der Gemeinsamen Außen- und Sicherheitspolitik (GASP) hinaus und betrifft sowohl die Außenhandels- als auch die Erweiterungspolitik als schärfste Machtinstrumente der EU (Meunier und Vachudova 2018). Beide werden zu stumpfen Schwertern, da sie zugleich die zentralen Mittel zum Export marktwirtschaftlicher und rechtsstaatlicher Normen sind. Auch die Erdgasimportpolitik ist von autokratischer Einflussnahme betroffen, wenn sich von außen induzierte staatskapitalistische Praktiken in der EU ausbreiten und bereits umgesetzte Liberalisierungsmaßnahmen zurückdrehen, wie der Verfasser darlegen wird.

Als erschwerender Faktor kommt hinzu, dass unter Trump auch die politische Rückendeckung für die EU passé ist. Strafzölle auf europäische Stahlimporte und der amerikanische Rückzug aus dem von der EU maßgeblich ausgehandelten Nuklearabkommen mit dem Iran stehen exemplarisch für den „harten Einbruch weltpolitischer Realitäten“ (Speck 2019), dem die Europäische Union ausgesetzt ist. In diesem Umfeld verstricken sich manche Mitgliedstaaten der EU zunehmend in einer inneren Mächtekonkurrenz und buhlen um die Gunst autokratischer Großmächte. China nutzt diese Lage strategisch zur Verbreitung der eigenen Staats- und Wirtschaftsordnung, indem es die nationalen Sonderwege zusätzlich befeuert. Internetkrieger im Dienste Russlands verbreiten in den sozialen Medien Desinformationen zur Zersetzung des öffentlichen Diskurses, um auf diese Weise in der EU nationalistische, EU-feindliche Parteien heranzuzüchten (Orenstein und Kelemen 2017), die es auch in Mitgliedstaaten ohne Systemtransformation nach 1990 wie Italien und Österreich zeitweise zu Regierungsbeteiligungen gebracht haben. Auch die britischen Tories als etablierte Regierungspartei aus dem traditionellen Mitte-Rechts-Spektrum sind vor Interventionen des russischen Staates nicht gefeit: Zur Unterstützung der Brexit-Kampagne haben russische Trolle am Tag des Referendums auf Twitter tausende Nachrichten verschickt (Field und Wright 2018). Ungeklärte Verbindungen von Dominic Cummings, dem Kampagnendirektor von Vote Leave, zu russischen Sicherheits- und Geheimdienstkreisen werfen zusätzliche Fragen nach einer Einflussnahme des Kreml auf (Bennetts 2019; Harper und Wheeler 2019). Dieses Phänomen betrifft alte wie neue Mitgliedstaaten der Union. Daher hängt die außenpolitische Macht und Handlungsfähigkeit der EU in erster Linie vom Fortbestand demokratischer Staatspraxis im Innern ab, und nicht primär von ihrer räumlichen Ausdehnung, wie Herfried Münkler irrtümlich argumentiert (Münkler 2019). Freilich ist die innerstaatliche Garantierung einer demokratischen Verfassungswirklichkeit keine hinreichende Bedingung für ein effektives auswärtiges Handeln, das zusätzlich die Bereitschaft des Europäischen Parlaments zur außenpolitischen Mitwirkung erfordert, zumal unter den Abgeordneten weitgehend ein anti-totalitärer Konsens mit Blick auf außenpolitisch relevante Fragestellungen herrscht und der Kreml sowie die Kommunistische Partei Chinas nur wenige Anhänger haben (Krekó et al. 2020, S. 18).

## Im Klammergriff der Autokraten

Der Klammergriff der Autokraten von innen und außen erschwert die Erfüllung der Ziele in der EU-Erweiterungspolitik. Sie soll mit der verlockenden Aussicht auf die Mitgliedschaft in der EU das gesamte Unionsrecht in den Kandidatenländern einführen. Das Wunschergebnis: Junge Demokratien stabilisieren sich und verfestigen ihre soziale Marktwirtschaft. Die Frage, ob der machtpolitische Hebel eines EU-Beitritts weiterhin einsetzbar ist, stellt sich vor allem bei der bis 2025 angestrebten Westbalkan-Erweiterung. Denn in der Balkan-Frage bündeln sich diverse strategische Ziele unionsinterner und externer Akteure, die mit denen der EU nicht immer kompatibel sind. Einzig die Türkei scheint aus ökonomischen und sicherheitspolitischen Motiven daran interessiert, die Staaten in der Region in ihren NATO- und EU-Beitrittsplänen zu unterstützen (Aydıntaşbaş 2019). Dagegen investieren einige Golfstaaten und Saudi-Arabien in erheblichem Maße in Bildungs- und Erziehungseinrichtungen in der Region, um eine reaktionäre Lesart des Islams zu verbreiten (Lilyanova 2017).

Hinzu kommt, dass die Unionsinteressen von innen durch den ungarischen Ministerpräsidenten Viktor Orbán unterlaufen werden und Gelegenheitsstrukturen für andere politische Player schaffen. Aus der ungarischen Annäherung an das autokratische Regierungssystem Russlands kann der Kreml politisches und ökonomisches Kapital schlagen:

1. Der im März 2019 angekündigte Umzug der in Moskau ansässigen Entwicklungsbank International Investment Bank (IIB) nach Budapest ist ein Beleg für die Annäherung Ungarns an den Kreml – vor allem vor dem Hintergrund des andauernden Konflikts mit den EU-Institutionen. Die Internationale Investitionsbank versteht sich selbst als eine kleinere Version der Europäischen Bank für Wiederaufbau und Entwicklung. Im Gegenzug für die Verlegung der IIB nach Ungarn, hat Orbán sich bereit erklärt, der IIB eine breite Palette von Privilegien zu gewähren, darunter die vollständige Immunität vor jeglicher Regulierung und Strafverfolgung, den Diplomaten-Status für das Gebäude der Bank, die Mitarbeiter^1^ und Gäste. Unter den neun Anteilseignern der IIB befinden sich ehemals sozialistische EU-Staaten wie Ungarn, Rumänien und Bulgarien sowie Kuba und Vietnam, die weiterhin von kommunistischen Staatsparteien regiert sind. Gemeinsam halten die EU-Länder den größten Anteil an der Bank, Russland bleibt jedoch der größte Einzelaktionär (Seddon und Hopkins 2019).
2. Für Russland eröffnet zudem der Energiehandel mit Ungarn Ansatzpunkte zur Verbreitung eigener staatskapitalistischer Praktiken. Besonders eindrücklich für das Ausmaß von Russlands Staatseinfluss ist die vom Kreml zu 80 % finanzierte Erweiterung des ungarischen Kernkraftwerks Paks, Paks 2 (Butler 2018). Zur Verdeutlichung der Dimension des Projekts geben Deák und Weiner an, dass der Umfang der russischen Kreditlinie für Paks 2 Moskaus gesamte Direktinvestitionen in Ungarn etwa um das Dreifache übersteigt. Schöpfte die Orbán-Regierung das Darlehen voll aus, stünde sie gegenüber der russischen Regierung in Höhe von 10 % des ungarischen Bruttoinlandsprodukts in der Haftung (Deák und Weiner 2019).

Auch im Erdgasmarkt bereiten die Verstaatlichungspläne der Fidesz-Regierung russischen Wirtschaftsinteressen einen idealen Nährboden. Das Ziel, die alleinige Verantwortung für Gashandelsfragen auf Regierungsebene zu bündeln, hat Orbán in zwei Etappen umgesetzt: Der erste Schritt bestand darin, im Jahr 2011 durch einen Neuzuschnitt der MVM^2^ Group ein staatseigenes Unternehmen für den Energiehandel aufzubauen (Deák und Weiner 2019). In einem zweiten Schritt übernahm die Hungarian Gas Trade Limited (MFGK) als Mitglied der MVM Group im Oktober 2013 dann das im Privatunternehmen MOL AG angesiedelte ungarische Gashandelsgeschäft der deutschen E.ON AG, so dass die MVM als Partei in den laufenden Liefervertrag mit Gazprom einsteigen konnte (Deák und Weiner 2019). Als unausgesprochene Gegenleistung erhielt Orbán vom Exportmonopolisten Gazprom einen Rabatt auf russisches Erdgas, mit dessen Hilfe er seine Wiederwahl 2014 sichern konnte (Deák und Weiner 2019). Für Russland ist der Handel mit Erdgas also auch ein strategisches Instrument, um politische Verbündete in der EU zu fördern. Intensivieren Kooperationspartner wie Orbán ihre staatlichen Eingriffe, kommt dies Russlands Interessen entgegen, da Energieaußenpolitik im russischen Staatsverständnis ohnehin ein zentrales Element nationalstaatlicher Souveränität darstellt. Die Ausbreitung staatskapitalistischer Handlungsformen in Mitgliedstaaten der EU verschafft Russland insoweit einen Machtvorteil gegenüber der EU, deren Gründungszweck auch darin besteht, Monopole durch Liberalisierungsmaßnahmen aufzubrechen. Noch effektiver könnte Russland das Energiehandelsgeschäft Gazproms in der EU als außenpolitischen Hebel einsetzen, wenn sich auch die Betreiberunternehmen von Erdgas-Speichern nicht mehr in privater Hand befänden. Bei der Bevorratung von Erdgas ist der Staat innerhalb der EU jedoch im Gegensatz zum Erdölsektor seltener als dominierender Akteur anzutreffen.

## Interessenkollision auf dem Balkan

Eine besorgniserregendere Folgewirkung der Expansion staatskapitalistischer Politikformen ist die empirische Beobachtung, dass je stärker sich in der EU korrupte Praktiken ausbreiten, supranational organisierte Außen- und Außenwirtschaftspolitik umso weniger effektiv gelingt, da externe Mächte durch die Ausnutzung oligarchischer Strukturen einzelne Integrationsmaßnahmen auf Ebene der Mitgliedstaaten blockieren oder umgehen. Innerhalb des föderal organisierten europäischen Staatenverbundes ist neben Ungarn – wie noch erläutert werden wird – vor allem Malta von dieser Entwicklung betroffen. Das Zusammenwirken externer und interner Kräfte erschwert mithin die Eindämmung von Korruption und stellt für supranationale EU-Institutionen ein Machtausübungsproblem gegenüber den eigenen Mitgliedstaaten (Integrationsmacht) und im Verhältnis zu außenpolitischen Partnern sowie Rivalen dar (Projektionsmacht).

Welch diffizile Angelegenheit die Erhaltung der unionseuropäischen Machtmittel des Binnenmarktzugangs sowie der territorialen und juristischen Expansion im Wege der Erweiterung sein kann, zeigt sich auf dem Westbalkan. In der Staatsorganisation und in den Wirtschaftssystemen der Beitrittskandidaten zur EU haben sich Oligarchie und Korruption festgesetzt. Auch in dieser Region leistet die Orbán-Regierung autokratische Entwicklungshilfe. So plant Orbán durch den bereits erfolgten Aufkauf von Presseorganen in Mazedonien und im EU-Mitgliedstaat Slowenien (Faktor 2020, S. 4-5) eine wesensverwandte Medienlandschaft zu schaffen (Kingsley 2018; Gergely 2019). Ziel der Maßnahmen ist es, das *illiberale* ungarische Staatsmodell in die Region zu exportieren (Jóźwiak und Żornaczuk 2018). Dem serbischen Präsidenten Aleksandar Vučić dienen die ungarischen Gleichschaltungsmaßnahmen in Presse und Rundfunk bereits als Vorbild (Csaky 2019). Auch russische Geheimdienste finden so auf dem Balkan günstige Bedingungen zur Diskreditierung der Erweiterungsbestrebungen von NATO und EU vor (Stronski und Himes 2019). Eine mögliche russische Unterstützung eines Putschversuchs gegen die Regierung in Montenegro im Oktober 2016, das seit Juni 2017 Mitglied der NATO ist, ist noch nicht abschließend geklärt (Martens 2019).

In Serbien konsolidiert sich Vučićs wenig demokratieaffiner Herrschaftsanspruch auch mit Hilfe chinesischer Überwachungstechnologie, die offen gegen EU-Standards verstößt (Stojkovski 2019). Dieser Fall belegt, wie Pekings Interventionen EU-Aspiranten vom Rechtsstaat zusätzlich entfremden können. Als Vehikel für Chinas machtpolitische Ambitionen auf dem Balkan und der Region Mittelosteuropa dient seit 2012 das regionale 17 + 1-Forum (Skorić 2019).^3^ An dem Format beteiligt sind die Visegrád-Staaten (Polen, Tschechien, Slowakei, Ungarn), die Westbalkan-Länder, seit 2019 auch Griechenland.^4^ Zu den bekanntesten Projekten der staatlichen China Road and Bridge Corporation gehören die geplante Pelješac-Brücke an der kroatischen Adria-Küste und die Schnellbahntrasse zwischen Belgrad und Budapest. In Bosnien finanziert China Projekte im Wert von 3,8 Mrd. €, welche vor allem im Energiesektor die Beihilfevorschriften der Europäischen Union missachten und die Aussicht auf den Beitritt des kleinen Staates mit einem BIP von 17 Mrd. € beeinträchtigen (Geinitz 2019). Das Beispiel der Interessenkollision auf dem Balkan zeigt: Unionsinterne und externe Autokraten müssen nicht notwendigerweise direkt zusammenarbeiten, um die Rechtsstaatsziele der EU-Erweiterungspolitik zu unterlaufen. Es reicht vollkommen aus, wenn beide Seiten mit wirkungsgleichen Maßnahmen an ähnlichen Zielen arbeiten. Gewinnen China und Russland mit Unterstützung Ungarns auf dem Balkan ungebrochen an Einfluss, wird die Aufnahme Serbiens, Mazedoniens und Bosnien-Herzegowinas voraussichtlich am Veto der rechtsstaatlich entwickelten EU-Mitgliedstaaten scheitern, da letztere Einspruch gegen die geplanten Beitritte einlegen werden. Auch über die Region hinaus wird die Erweiterungspolitik als außenpolitischer Hebel dann Schaden nehmen.

## Wachsende ökonomische Abhängigkeit von China in der EU-Handelspolitik

Die wachsende ökonomische Abhängigkeit von China birgt ein beträchtliches Risiko für die Schlagkraft der Gemeinsamen Handelspolitik (GHP) – einem kräftigen machtpolitischen Hebel, mit dem die EU auch Demokratieförderung betreibt. Denn im Gegenzug für den Zugang zum Binnenmarkt baut die EU auch Regeln zur verantwortungsvollen Staatsführung in die Abkommen und Zollvereinbarungen ein. Damit diese Maßnahmen zum Erfolg führen können, müssen die Regeln der Union zunächst im Binnenmarkt durchsetzbar sein. Ob es Italien wirklich gelingt, die eigene Teilnahme am One Belt, One Road-Projekt (OBOR) der Volksrepublik im Einklang mit den Wettbewerbsnormen der EU durchzusetzen, bleibt ungewiss. Das chinesische Kapital für industriepolitische Schlüsselprojekte wie für den Umbau der Häfen in Genua und Triest (Döhne 2019) gibt der Regierung in Peking ein zusätzliches politisches Druckmittel, um Italiens Position bei EU-Entscheidungen zu beeinflussen. Das gilt auch für die GHP. Ihre Kraft könnte stark nachlassen, wenn Peking künftig von ökonomisch abhängigen EU-Ländern bei Schlüsselentscheidungen ein Veto gegen *Good Governance*-Vorschriften einfordert, falls diese gegen chinesische Interessen verstoßen. Zwei nichthandelspolitisch gelagerte Fälle aus der jüngeren Vergangenheit belegen dieses Risiko: So hat Griechenland im Juni 2017 eine gemeinsame EU-Erklärung mit Kritik am Grundrechtsschutz in China im UN-Menschenrechtsrat blockiert (Emmott und Koutantou 2017). Ungarn hat sich einer kritischen Stellungnahme der EU-Botschafter in China zur OBOR-Initiative nicht angeschlossen (Heide et al. 2018). Beide Staaten gehören zu den größten Empfängern chinesischer Direktinvestitionen in der EU.

Ein derartiges Ausscheren einzelner EU-Länder würde vor allem das demokratiefördernde und marktwirtschaftliche Wirkungsvermögen der GHP gegenüber Entwicklungsländern schwächen, in denen die EU und China häufig um Einfluss konkurrieren. Die Europäische Union gewährt denjenigen Staaten, die nicht mit ihr über ein Freihandelsabkommen verbunden sind, Zollermäßigungen bis hin zu Zollfreiheit im Gegenzug für – anhand der Umsetzung von UN-Konventionen – nachprüfbare rechtsstaatliche Fortschritte (Bundesministerium für Wirtschaft und Energie 2020). Das handelspolitische Instrument hierfür nennt sich APSplus, das im Rahmen des Allgemeinen Präferenzsystems (APS) der EU angewendet wird. Zu den Begünstigten zählen aktuell Armenien, Bolivien, Kap Verde, Kirgisistan, Mongolei, Pakistan, die Philippinen und Sri Lanka (Europäische Kommission 2019).

Als Teil des APS gewährt die Europäische Union Entwicklungsländern eine Präferenzbehandlung im Rahmen der Regelung Alles außer Waffen (Everything But Arms, EBA). Diese kann die EU zur Konditionierung handelspolitischer Vorzüge einsetzen. Ein exemplarischer Anwendungsfall ist Kambodscha. Hier hat die Europäische Kommission am 12. Februar 2020 den zoll- und kontingentfreien Zugang des südostasiatischen Landes zum EU-Binnenmarkt aufgrund schwerwiegender und systematischer Menschenrechtsverletzungen teilweise zurückgenommen. Der vom ehemaligen Handelskommissar Phil Hogan und dem Außenbeauftragten Josep Borrell verkündete Beschluss betrifft die Aufhebung des präferenziellen Zugangs zum Binnenmarkt für rund 20 % der kambodschanischen Ausfuhren in die EU im Wert von 1 Mrd. € in Form von Kleidung, Schuhen, Reiseartikeln und Zucker (Europäische Kommission 2020a). Kambodscha darf diese Waren zwar weiterhin in die EU exportieren, jedoch unterliegen die Güter den allgemeinen Zollsätzen der Welthandelsorganisation. Da das Europäische Parlament und der Rat keine Einwände gegen die Maßnahme erhoben haben und der Sanktionsgrund innerhalb von sechs Monaten nicht entfallen ist, konnte der Kommissionsbeschluss am 12. August 2020 in Kraft treten (Europäische Kommission 2020b).

Der Umstand, dass Chinas OBOR-Initiative nicht nur auf *illiberale* Regierungen zielt, sondern beispielsweise auch in Deutschland Anwendung findet^5^, relativiert jedoch nicht die Bedeutung der inneren Regimefrage für die EU. Denn Finanzmittel aus China und Russland können existierende regierungsnahe oligarchische Machtstrukturen wie in Ungarn, Malta oder Zypern festigen oder ihre Entwicklung begünstigen. Staaten, in denen wirtschaftliche Macht in den Händen weniger Akteure konzentriert ist, weisen eine hohe Wesensverwandtschaft zum staatskapitalistischen Wirtschaftsmodell aus Fernost auf. Damit das Wirtschaftsmodell in der EU keine Schule macht, muss die Union verhindern, dass China es oligarchisch organisierten oder weniger finanzkräftigen EU-Ländern mit Hilfe der OBOR-Initiative schrittweise aufstülpt. Denn auch eine Ausbreitung neomerkantilistischer Strukturen untergräbt eine funktionsfähige wie auch glaubwürdige Erweiterungs- und Außenhandelspolitik der Union. Der im April 2019 vereinbarte EU-Mechanismus zur Prüfung ausländischer Direktinvestitionen (ADI) (Europäische Union 2019) ist daher ein notwendiger Schritt, um die ADI aus Drittstaaten zu überwachen. Ein Beleg: Allein zwischen 2016 und 2017 ist der Investitionsanteil chinesischer Staatsbetriebe an Pekings Gesamtinvestitionen in der gesamten EU von 35 % auf 68 % angewachsen (Hanemann und Huotari 2018). Es ist damit zu rechnen, dass diese Entwicklung auch eine potenziell von der Covid-19-Pandemie ausgelöste punktuelle Rückverlagerung von globalen Wertschöpfungsketten wie etwa in der Gesundheitswirtschaft überdauern wird.

## Kontrolllücken in der supranationalen Energiegesetzgebung

Dass autokratische Einflussnahme von außen nicht nur von Großmächten wie China betrieben wird oder allein die postsozialistischen Staaten tangiert, demonstriert das Vorgehen Aserbaidschans auf Malta. Wie im Falle der Energiebeziehungen Ungarns zu Russland bereits dargelegt, haben sich auch im Inselstaat Großprojekte zur Einfuhr von Energieträgern als besonders verwundbar für externe illegale Interventionen erwiesen. Beim Bau des schwimmenden Gaskraftwerks Delimara und der Aushandlung eines unwirtschaftlichen Liefervertrags (Borg 2018) haben maltesische Eliten mit dem aserischen Staatsunternehmen Socar oligarchische Herrschaftspraktiken kultiviert, die in einem politischen Mord an der Journalistin Daphne Caruana Galizia kulminierten. Die investigative Reporterin musste mit dem Leben bezahlen, da sie überhöhte Abnahmegarantien und Haftungsrisiken für den Staat im Kontext des betreffenden Energiegeschäfts aufgedeckt und darüber hinaus wiederholt Korruptionsfälle maltesischer Spitzenpolitiker publik gemacht hatte (Caruana Galizia 2017). Ihr Tod hat die Pressefreiheit auf Malta massiv in Frage gestellt und die Dringlichkeit von außenpolitischen Gegenmaßnahmen der EU zur Bekämpfung von importierter Korruption vor Augen geführt. Wie aus Akten hervorgeht, die der investigativen Journalistin bereits zu Lebzeiten zugespielt worden waren, ist die Regierung Maltas in einem Vertrag mit Socar umfangreiche Verpflichtungen eingegangen: Die sozialdemokratische Regierung des damals amtierenden Ministerpräsidenten Joseph Muscat hat darin zugesagt, den gesamten Erdgasbedarf für die Versorgung der eigenen Kraftwerke im Wert von mehr als einer Milliarde € zu einem Fixpreis mit einer Laufzeit von zehn Jahren ab 2015 aus Aserbaidschan zu beziehen (Garside 2018). Seit Lieferbeginn im April 2017 profitiert Socar somit von der Diskrepanz zwischen dem vertraglich fixierten und einem fast konstant niedrigeren Marktpreis.

Integrationspolitisch hat der Vertrag über verflüssigtes Erdgas (LNG) eine Kontrolllücke der supranationalen Unionsgesetzgebung offenbart. Diese manifestiert sich insoweit, als dass die Energieunion-Gesetzgebung der EU keine expliziten Anti-Korruptionsvorschriften verankert hat. Das gilt sowohl für den EU-Beschluss 2017/684 über die ex ante-Prüfung zwischenstaatlicher Energieabkommen als auch für die Security of Supply-Verordnung 2017/1938 der EU zur Prävention von Risiken für die Versorgungssicherheit (Stüwe 2020).

Weiteren Handlungsspielraum erhalten regierungsnahe und regierende Oligarchen durch zwei Strukturmerkmale der Gasimportpolitik: Zum einen durch die verbreitete Praxis, kommerzielle Lieferverträge durch zwischenstaatliche Abkommen rechtlich abzusichern, und zum anderen aufgrund der kostenintensiven Transportinfrastruktur wie Pipelines oder Häfen zur lieferfähigen Umwandlung von LNG (Stüwe 2020).

## Maßnahmen zur Behebung von Rechtsstaatsdefiziten einzelner Mitgliedstaaten

Da die zentralen europäischen Machtmittel der Außenhandels- und Erweiterungspolitik gegenüber China und Russland eine begrenzte Wirkung haben, muss die EU die Rechtsstaatsdefizite einzelner Mitgliedstaaten möglichst *kurzfristig* beheben und so die Regimefrage im Inneren klären. Vier Maßnahmen sollte sie prioritär ergreifen:

Damit die Zangenbewegung aus externen und internen Autokraten die wenigen außenpolitischen Stärken der EU nicht nachhaltig lähmt, braucht die EU-Kommission ein Mandat zur Bündelung einzelner, themenspezifischer Vertragsverletzungsverfahren, falls eine systemische Gefährdung der Rechtsstaatlichkeit auftritt.
a) Die beim EU-Sondergipfel vom 17. bis 21. Juli 2020 diskutierte Verankerung automatischer Kürzungen von EU-Programmgeldern im mehrjährigen Finanzrahmen (MFR) der EU zwischen 2021-2027 und im verabschiedeten Corona-Aufbaufonds hat aufgrund des Zwangs zur Einstimmigkeit im Europäischen Rat zunächst nur eine geringe Aussicht, politische Wirklichkeit zu werden. In den Schlussfolgerungen des Gipfels ist allerdings in Nr. 23 die Zusage enthalten, dass eine „Konditionalitätsregelung zum Schutz des Haushalts und von Next Generation EU eingeführt“ (Europäischer Rat 2020) wird, verbunden mit einer Aufforderung an die Kommission, im Fall von Verstößen, Maßnahmen vorzuschlagen, die vom Rat mit qualifizierter Mehrheit angenommen werden. Dieser Passus lässt sich als Plädoyer lesen, die Verabschiedung des bereits existierenden Verordnungsvorschlags vom 2. Mai 2018, den noch die Juncker-Kommission vorgelegt hatte, zur Bindung der Struktur- und Kohäsionsförderung an rechtsstaatliche Prinzipien voranzutreiben. In der Tat hätte dieser eine bessere Chance, den Rat und das Europäische Parlament zu passieren, da das geltende Ordentliche Gesetzgebungsverfahren in beiden Legislativkammern einen Mehrheitsentscheid über den Rechtsakt erlaubt (Europäische Kommission 2018). Im Rahmen der im Oktober 2020 noch anhaltenden informellen Trilog-Verhandlungen hat die deutsche Ratspräsidentschaft die womöglich einmalige Chance, einen kraftvollen Rechtsstaatsmechanismus im Haushaltsrecht der EU zu verankern und diesen separat vom einstimmig zu beschließenden MFR zu verabschieden. Denn aufgrund des Wirtschaftseinbruchs infolge der Covid-19-Pandemie sind die rechtsstaatlich destruktiven Regierungen in Polen und Ungarn auf die Finanzmittel des MFR sowie des Aufbaufonds angewiesen. Vetodrohungen aus Budapest und Warschau gegen den für MFR und Aufbaufonds erforderlichen Eigenmittelbeschluss des Rates sind daher im Kern als Bluff zu werten.
  b) Ein anderer Vorschlag zur Ahndung von Rechtsstaatsverstößen stammt aus der Feder der European Stability Initiative (ESI) – einem internationalen Think Tank. Demnach sollten sich Parlament und Mitgliedstaaten darauf einigen, EU-Finanzsanktionen dann zu verhängen, wenn eine Regierung sich weigert, ein EuGH-Urteil umzusetzen, in dem ein Verstoß gegen Art. 19 des EU-Vertrags festgestellt wurde, was bislang in zwei Vertragsverletzungsverfahren gegen Polen geschah (ESI 2020, S. 2‑3.). Art. 19 des Vertrages über die Europäische Union - ein Eckpfeiler der Unionsrechtsordnung - verpflichtet die Mitgliedstaaten, die erforderlichen Rechtsbehelfe zu schaffen, damit ein wirksamer Rechtsschutz in den vom Unionsrecht erfassten Bereichen gewährleistet ist.
Damit sich kleptokratische Strukturen in der EU nicht verfestigen, müssen die 22 Teilnehmerstaaten der Europäischen Staatsanwaltschaft (EUStA) zudem den Druck auf Ungarn und Polen erhöhen, der EUStA beizutreten. Als unabhängige Einrichtung der EU ist die EUStA im Rahmen der Verstärkten Zusammenarbeit gemäß Art. 86 des Vertrages über die Arbeitsweise der Europäischen Union (AEUV) zur Bekämpfung des Missbrauchs von Finanzmitteln der Union gegründet worden. Ein Mandat in allen Mitgliedstaaten gäbe ihr eine implizite außenpolitische Bedeutung, da sie autokratisch gesinnten EU-Regierungen Möglichkeiten nähme, im Konzert mit externen Großmächten die Aushöhlung der Rechtstaatlichkeit voranzutreiben.
Zudem sollten alle europäischen Parteien ihre nationalistischen Mitglieder wie den ungarischen Fidesz, die rumänische Partidul Social Democrat (PSD) und die tschechische ANO aus den eigenen Reihen ausschließen, um nicht selber in ein autokratisches Fahrwasser zu geraten. Die erklärte sogenannte Suspendierung des Fidesz aus der Europäischen Volkspartei (EVP) und der PSD aus der Sozialdemokratischen Partei Europas (SPE) (Becker 2019) hat der Zusammenarbeit jedoch kein unmittelbares Ende gesetzt. Das lässt sich insbesondere im Europäischen Parlament beobachten. Hier vertreten Fidesz- und PSD-Abgeordnete weiterhin ihre Parlamentsfraktionen in den Ausschüssen und nehmen an gruppeninternen Abstimmungen teil. Die *Suspendierung *ist insofern primär als ein taktisches Manöver zu sehen und sollte nicht als eine echte Kündigung der Mitgliedschaft in der Europapartei missverstanden werden.

## Kapazität zur *legal warfare*

Zur Verwirklichung der in Kap. 5 und 6 genannten Maßnahmen hat die abgeschlossene personelle und programmatische Konstituierungsphase der EU-Institutionen im Jahr 2019 ihre Erwartungen allenfalls partiell einlösen können, obwohl Kommissionspräsidentin von der Leyen und ihre designierten EU-Kommissare Didier Reynders und Vera Jourová ihre Anhörungen vor dem Europäischen Parlament nicht ohne programmatische Zusagen in Sachen Rechtsstaatlichkeit an die Fraktionen der Liberalen und der Grünen bestehen konnten.

Was die Stärkung des Rechtsstaatsprinzips im auswärtigen Handeln der Union anbelangt, blieb insbesondere die Ressortverteilung an die EU-Kommissare hinter den Erwartungen zurück. Nach der Ablehnung des ersten ungarischen Kandidaten László Trócsányi mussten die Parlamentarier die bittere Pille schlucken, dass sich die Orbán-Regierung mit der Nominierung Olivér Várhelyis den faktischen Zugriff auf die demokratiesensible Erweiterungs- und Nachbarschaftspolitik sichern konnte und sich die dargelegten Missstände auf dem Westbalkan daher zu perpetuieren drohen. Außenpolitisch kam die von der Leyen-Kommission insoweit mit einer personellen Hypothek ins Amt. Den Fraktionen der liberalen Renew Europe^6^ und der Grünen/EFA war es trotz ihrer erheblichen Stimmengewinne bei der Wahl (Liberale +39, Grüne +23) in dieser Hinsicht nicht vergönnt, ihre neu erlangte Rolle als Königsmacher der Kommission in unmittelbaren außenpolitischen Einfluss umzuwandeln. Dennoch kann das Europäische Parlament, wie Andreas Marchetti zu Recht anmerkt, über Ziele, Prioritäten und Budgetausstattung von Einzelinstrumenten europäischer Außenpolitik im Ordentlichen Gesetzgebungsverfahren mitentscheiden (Marchetti 2019, S. 12). Ein solcher Machtzuwachs ist insbesondere für die Legislaturperiode 2019-2024 absehbar, da die konservative EVP und die sozialdemokratische Fraktion der Progressiven Allianz der Sozialdemokraten (S&D) ihre gemeinsame Mehrheit bei der Wahl zum Europäischen Parlament verloren haben. Auch dass die erforderliche qualifizierte Mehrheit für Ursula von der Leyen im Europäischen Rat nicht ohne die zum damaligen Zeitpunkt vorhandenen sieben Stimmen der liberalen Regierungschefs (Zentrum für Europäische Integrationsforschung 2020) zustande kommen konnte, erwies sich nicht als hinreichendes Druckmittel, um das Erweiterungsressort mit einem demokratiepolitisch unbedenklichen Amtsanwärter zu besetzen, da die Kommissionspräsidentin bei ihrer Wahl im Europäischen Parlament wohl auf die Stimmen der Fidesz-Partei angewiesen war. Für die Entscheidungsträger der EU wird es im weiteren Verlauf der Legislaturperiode daher zunächst entscheidend darauf ankommen, nach der Finalisierung des Corona-Aufbaufonds und des MFR 2021-2027 die demokratische DNA der Europäischen Union mit der Verabschiedung des von der Juncker/Timmermans-Kommission 2018 vorgelegten Rechtsstaatsmechanismus zu schützen.

Mittel- bis langfristig müsste die EU allerdings eine gemeinschaftliche Art der politisch-juristischen Machtprojektion entwickeln oder in den Worten Steven Blockmans’ eine Kapazität zur *legal warfare* erlangen, die über bisherige Einflusskanäle wie die Erweiterungs- und Nachbarschaftspolitik hinausginge. Anders als es die martialische Wortschöpfung nahelegt, verstieße eine solche Außenpolitik weder gegen nationales noch internationales Recht. Auch würde nach Auffassung von Blockmans eine solche Außenpolitik keiner hyperaggressiven Praxis Vorschub leisten, um die Interessen der EU rücksichtslos zu fördern. Machtinstrumente der Wahl wären eine Kombination aus internationalem, EU- und nationalem Recht sowie multilateralen Foren. Auf diese Weise könnte die EU unter Ausnutzung ihrer Marktmacht die Kosten illegaler Praktiken von internationalen Rivalen erheblich erhöhen (Blockmans 2020). Das Ziel kann also nur eine europäische Umwidmung des Begriffs amerikanischer Provenienz sein. Zu den weiteren *Lawfare*^7^-Taktiken würden die gezielte Einleitung von Untersuchungen durch internationale Organisationen oder die Einberufung von Abstimmungen in der UN-Generalversammlung sowie vor internationalen Schiedsgerichten bei Handelsstreitigkeiten gehören. Auch gesetzgeberische Maßnahmen auf Unionsebene wie die Verschärfung von Vorschriften, um etwa Banken und Energieunternehmen daran zu hindern, Diktaturen und deren Kunden zu bedienen oder zum Schutz von europäischen Unternehmen vor sekundären US-Sanktionen wären Teil eines solchen Fähigkeitsprofils (Blockmans 2020).

## Fazit

Es bleibt festzustellen, dass die auswärtigen Politiken der EU den Faktor Rechtstaatlichkeit nicht außer Acht lassen dürfen. Die vorliegend erörterten Politikfelder der Außenhandels‑, Energieimport- und Erweiterungspolitik bieten zahlreiche Beispiele für verpasste Chancen. Doch eine derartige Lethargie kann sich die EU in einem rauen Umfeld globaler Mächtekonkurrenz politisch nicht länger erlauben. Damit ihre schärfsten Machtmittel nicht stumpf werden, muss sie ihre innere rechtsstaatliche Verfasstheit im aufgeklärten Eigeninteresse erhalten und in den Außenbeziehungen als handlungsleitendes Prinzip effektiv zur Geltung bringen.

## Footnotes

1 Im vorliegenden Artikel wird, abweichend vom ZfAS-Standard, bei personenbezogenen Substantiven die männliche grammatikalische Form verwendet. Der Autor schließt damit Personen jeden Geschlechts gleichermaßen ein.

2 Magyar Villamos Művek. Zu Deutsch: Ungarische Elektrizitätswerke.

3 Zu den 17 Staaten gehören Albanien, Bosnien-Herzegowina, Bulgarien, Griechenland, Kroatien, die Tschechische Republik, Estland, Ungarn, Lettland, Litauen, Mazedonien, Montenegro, Polen, Rumänien, Serbien, Slowakei und Slowenien.

4 Bereits im Jahr 2016 hatte die *volkseigene* China COSCO Shipping Corporation den Hafen von Piräus übernommen.

5 Auch in Deutschland befürchten Kritiker seit der Übernahme des deutschen Roboterherstellers Kuka 2016 durch die chinesische Midea Group eine Verlagerung der industriellen Wertschöpfung nach Fernost (Brüggmann 2016).

6 Renew Europe ist die Nachfolgerin der Allianz der Liberalen und Demokraten für Europa (ALDE).

7 Der Begriff Lawfare wurde 2001 in den Vereinigten Staaten geprägt, die das Finanz‑, Versicherungs- und Energierecht wirksam eingesetzt haben, um ihre Macht zu vergrößern und die ihrer Gegner zu kontrollieren (Blockmans 2020).
